# Evolutionary conserved microRNAs are ubiquitously expressed compared to tick-specific miRNAs in the cattle tick *Rhipicephalus (Boophilus) microplus*

**DOI:** 10.1186/1471-2164-12-328

**Published:** 2011-06-24

**Authors:** Roberto A Barrero, Gabriel Keeble-Gagnère, Bing Zhang, Paula Moolhuijzen, Kazuho Ikeo, Yoshio Tateno, Takashi Gojobori, Felix D Guerrero, Ala Lew-Tabor, Matthew Bellgard

**Affiliations:** 1 Centre for Comparative Genomics, Murdoch University, WA 6150, Australia; 2 Department of Employment, Economic Development and Innovation (DEEDI) Biotechnology Laboratories, The University of Queensland, St Lucia, QLD 4067, Australia; 3 CRC for Beef Genetic Technologies, University of New England, Armidale, NSW 2351, Australia; 4 Center for Information Biology and DNA Databank of Japan, National Institute of Genetics, Yata 1111, Mishima, Shizuoka 411-8540, Japan; 5 US Department of Agriculture, Agricultural Research Service, 2700 Fredericksburg, Rd., Kerrville, TX 78028, USA; 6 Queensland Alliance for Agriculture and Food Innovation Institute, The University of Queensland, c/o DEEDI, St Lucia, QLD 4067, Australia

## Abstract

**Background:**

MicroRNAs (miRNAs) are small non-coding RNAs that act as regulators of gene expression in eukaryotes modulating a large diversity of biological processes. The discovery of miRNAs has provided new opportunities to understand the biology of a number of species. The cattle tick, *Rhipicephalus *(*Boophilus*) *microplus*, causes significant economic losses in cattle production worldwide and this drives us to further understand their biology so that effective control measures can be developed. To be able to provide new insights into the biology of cattle ticks and to expand the repertoire of tick miRNAs we utilized Illumina technology to sequence the small RNA transcriptomes derived from various life stages and selected organs of *R. microplus*.

**Results:**

To discover and profile cattle tick miRNAs we employed two complementary approaches, one aiming to find evolutionary conserved miRNAs and another focused on the discovery of novel cattle-tick specific miRNAs. We found 51 evolutionary conserved *R. microplus *miRNA loci, with 36 of these previously found in the tick *Ixodes scapularis*. The majority of the *R. microplus *miRNAs are perfectly conserved throughout evolution with 11, 5 and 15 of these conserved since the Nephrozoan (640 MYA), Protostomian (620MYA) and Arthropoda (540 MYA) ancestor, respectively. We then employed a de novo computational screening for novel tick miRNAs using the draft genome of *I. scapularis *and genomic contigs of *R. microplus *as templates. This identified 36 novel *R. microplus *miRNA loci of which 12 were conserved in *I. scapularis*. Overall we found 87 *R. microplus *miRNA loci, of these 15 showed the expression of both miRNA and miRNA* sequences. *R. microplus *miRNAs showed a variety of expression profiles, with the evolutionary-conserved miRNAs mainly expressed in all life stages at various levels, while the expression of novel tick-specific miRNAs was mostly limited to particular life stages and/or tick organs.

**Conclusions:**

Anciently acquired miRNAs in the *R. microplus *lineage not only tend to accumulate the least amount of nucleotide substitutions as compared to those recently acquired miRNAs, but also show ubiquitous expression profiles through out tick life stages and organs contrasting with the restricted expression profiles of novel tick-specific miRNAs.

## Background

The Arthropods are a diverse group of organisms including Chelicerata (ticks, spiders), Myriapoda (centipedes, millipedes), Crustacea (crabs, shrimps), and Insecta (flies, beetles). Molecular estimates indicate that ticks emerged 300 ± 27 MYA, while the prostriate and metastriate hard tick lineages diverged 241 ± 28 MYA [1]. *Rhipicephalus *(*Boophilus*) *microplus *is considered to be the most economically important tick parasite in the world. *R. microplus *is a hard tick associated with cattle infestations but can also occasionally be found on other hosts including horses, goats, sheep, pigs and some wild animals living in subtropical and tropical regions worldwide [2,3].

*R. microplus *is a member of the metastriate lineage of ticks that includes numerous genera and species of medical and veterinary importance. In comparison, *I. scapularis *is a member of the prostriate lineage that comprises the single genus *Ixodes*. The prostriate and metastriate lineages differ markedly in many aspects of their biology such as type of developmental cycle (i.e., three-versus one-host ticks), host range and vector competence. Comparative analyses between prostriate and metastriate gene sets including miRNAs may reveal the genetic basis for fundamental differences in the biology of these tick lineages [4].

*R. microplus *is generally a single host tick spending all parasitic life cycle stages on cattle. The eggs hatch in the environment and the larvae crawl up grass or other plants to find a host. In the summer, *R. microplus *can survive for as long as 3 to 4 months without feeding. In cooler temperatures, they may live without food for up to six months. Newly attached seed ticks (larvae) are usually found on the softer skin inside the thigh, flanks, and forelegs. After feeding, the larvae molt twice, to become nymphs and male or female adults. Each developmental stage (larval, nymph and adult) feeds only once, but the feeding takes places over several days. Adult male ticks become sexually mature after feeding, and mate with feeding females. An adult female tick that has fed and mated detaches from the host and deposits a single batch of many eggs in the environment. Typically, these eggs are placed in crevices or debris, or under stones. The female tick dies after ovipositing. Ticks in the subgenus *Boophilus *have a life cycle that can be completed in 3 to 4 weeks; this characteristic can result in a heavy tick burden particularly on tick susceptible cattle in tropical areas [2,3].

MicroRNAs (miRNAs) are small 19--25 nucleotide regulatory RNAs that act as post-transcriptional modulators of gene expression in animals and plants [5]. They are estimated to represent 1% of the transcriptome in higher eukaryotes and predicted to control the expression of up to 30% of messenger RNAs [6,7]. Most miRNAs are encoded in intergenic regions and are transcribed by RNA polymerase II as long primary nuclear miRNAs (pri-miRNAs), which range from hundreds to thousands of nucleotides in length [8]. One pri-miRNA typically contains a single or several miRNA precursors (pre-miRNAs) as stem-loop, hairpin structures flanked by unstructured, single stranded RNA sequences [9]. Pre-miRNAs are cleaved near their loops by the cytoplasmic RNase III enzyme Dicer to generate a heteroduplex of two ~23-nt RNAs that are then packed into the RISC complex [10]. Mature miRNA sequences are encoded either in the 5'-arm or the 3'-arm of pre-miRNAs. The decision as to which sequence is incorporated into the silencing complex is influenced by the difference in pairing stabilities between the two ends of the miRNA:miRNA star (miRNA*) duplex, with preferential incorporation of the strand whose 5'end is less stably paired [11,12]. In some cases both strands of the miRNA:miRNA* duplex were found expressed at similar levels [13]. About half of the miRNA genes in *Drosophila melanogaster *are clustered and transcribed from a single polycystronic pri-miRNA [14].

Recently, next generation sequencing technologies have been utilized to profile and discover miRNAs genome-wide. To assist in this process it is normally required to have a reference genome sequence. Currently, there is no reference genome sequence for *R. microplus*, with the *Ixodes scapularis *genome draft (IscaW1.1) the closest reference genome in which 37 miRNAs have been identified [15]. Some miRNAs are highly conserved throughout evolution including let-7, present in metazoan lineages such as arthropods and vertebrates that diverged 641-686 MYA [16]. Thus, the use of the *D. melanogaster *reference genome for which 152 miRNAs are currently annotated [15] may allow not only the identification of highly conserved tick miRNAs, but also to discover arthropod-specific miRNAs. Previous studies have shown that miRNAs are continuously being added to metazoan genomes through time, and once these are integrated into gene regulatory networks, show only rare nucleotide substitutions within the mature miRNA sequence at predictable positions and are only rarely secondarily lost [17-19]. This is likely related to the strong purifying selection against changes in secondary structure of pre-miRNAs [20]. Wheeler and colleagues [21] documented evolutionary stable shifts to the determination of position 1 of the mature sequence that can be displaced towards either the 5' or 3' end, a phenomenon called seed shifting, as well as the ability to post-transcriptionally edit the 5' end of the mature read, changing the identity of the seed sequence and possibly the repertoire of downstream targets.

Currently there are no known microRNAs reported for *R. microplus *and we aimed at the identification and discovery of evolutionary conserved as well as novel tick-specific miRNAs in *R. microplus *by using a combination of next generation high throughput sequencing, comparative genomics and de novo computational screening. We constructed eight small transcriptome libraries derived from various cattle tick life stages and from selected organs including gut, salivary glands and ovaries. We aim to characterize changes in gene set and expression levels of miRNAs at various tick life stages as well as selected organs. We also conducted an evolutionary analysis to identify subsets of *R. microplus *miRNAs and miRNA* sequences that are perfectly conserved since either the Nephrozoa (641-686 MYA), Protostomia (618-653 MYA), Arthropoda (~540 MYA) or Ixodidae (~241MYA) ancestor [1,16,21]. We also provide evidence of seed shifts and gene duplications unique to the *R. microplus *lineage.

## Results and discussion

### Identification of evolutionary conserved and cattle tick-specific miRNAs

Currently there are 37 known miRNAs in *Ixodes scapularis*, a species belonging to prostriate hard tick lineage [15,21], but there are no known miRNAs identified for *R. microplus *or other metastriate hard tick species. In order to identify *R. microplus *miRNAs and expand the repertoire of cattle tick miRNAs and to obtain insights into changes in miRNA expression throughout the cattle tick life stages and in selected adult female tick organs, a high throughput sequencing approach was conducted. This approach generated more than 35 million short reads derived from the tick small RNA transcriptome from eggs, unfed larvae, larvae exposed for six hours to the host without being allowed to feed (frustrated larvae), and adult ticks as well as selected adult female tick organs (Table 1). To identify tick miRNAs we anticipated that a fraction of the known Arthopoda miRNAs should be conserved in both the Chelicerata (cattle ticks) and Insecta lineages regardless of their estimated divergence time of more than 500 MYA [16,21]. Under this assumption, sequenced short reads from tick samples could be mapped onto the *D. melanogaster *genome in order to identify identical or nearly identical conserved tick miRNAs. To be able to distinguish true sequence polymorphisms from non-specific mapping artefacts the performance of several short read aligners were initially evaluated to define the tool that mapped the largest amount of true positives and introduced a limited amount of false positive aligned short reads. We created simulated 36-bp short reads using mutation rates from 0.1% to up to 16% (Additional file 1) containing both SNPs and/or insertion/deletions (indels) and evaluated the ability of each tool to correctly align simulated short reads. Our results indicated that Novoalign produced the best overall short read mapping performance (Additional file 2). Thus, we used this tool to align the generated tick short reads onto the Drosophila genome. To ensure reliable short read alignments base quality scores were taken into account to conduct an interative alignment approach aiming to identify the best mapping position for each read. Novoalign generates a mapping quality score for each aligned read so that ambiguously mapped short reads can be removed from downstream analyses. Out of 35 million short reads derived from various cattle tick life cycle stages and three key organs, 3.5 million reads were aligned onto known miRNA loci on the Drosophila genome with at least a quality alignment score of Q = 1 (Table 1). We manually inspected the aligned reads onto miRNA loci and removed non-specific alignments. To identify possible duplicated *R. microplus *miRNAs all reads mapped onto a single *D. melanogaster *miRNA locus were inspected and if miRNA isoforms were observed these were required to be cloned in at least two distinct libraries to be validated. This approach identified 46 *R. microplus *miRNAs including four duplicated copies for rmi-let-7 (a, f, m and n), and two duplicated copies for rmi-miR-219 and rmi-miR-285. In addition we also detected the expression of 7 miRNA* sequences, of these four have counterparts in *D. melanogaster *[15] (Additional file 3).

**Table 1:** Short read statistics for evolutionary conserved and novel tick-specific *R. microplus *miRNAs

Sample	Total reads	Evolutionary conserved miRNAs	Novel tick-specific miRNAs	Total miRNAs
**A) Life cycle stages**				
Eggs	4,215,404	8,775	868	9,643
Larvae	3,964,440	732,690	39,959	772,649
Frustrated Larvae	5,473,363	628,561	31,617	660,178
Female	4,248,307	996,324	1,398	997,722
Male	4,071,427	344,877	683	345,560
sub total	21,972,941	2,711,227	74,525	2,785,752
**B) Female tick organs**				
Gut	3,501,156	413,240	1,028	414,268
Salivary glands	4,579,483	257,096	1,898	258,994
Ovaries	5,206,221	120,485	2,833	123,318
sub total	13,286,860	790,821	5,759	796,580
Total	35,259,801	3,501,629	80,287	3,582,332

It has been recently reported that there has been gain and loss of miRNA families in Arthropod lineages [21]. Thus some known miRNAs may have been lost in the *D. melanogaster *genome but still be present in tick genomes. To evaluate this possibility and also to identify novel tick-specific miRNAs we conducted a computational screening using MiRDeep as previously described [22]. To screen for candidate miRNA loci we aligned small RNA reads from each tick library onto both the *I. scapularis *draft genome (IscaW1.1; 369,492 contigs) and *R. microplus *draft genomic contigs that were recently sequenced and assembled by our group (Bellgard et al. unpublished data; 175,226 contigs encoding a total of 144,709,321 bp). This identified candidate pre-miRNA sequences that were further screened against nucleotide databases and those having similarity to known coding/non-coding genes were excluded. Furthermore, short reads from all libraries were then aligned onto remaining pre-miRNA candidates and those not having typical miRNA alignments were removed [23]. This analysis identified 44 and 25 miRNAs in *I. scapularis *and *R. microplus *genomic contigs, respectively. We next screened the identified miRNAs against all annotated miRNAs [15] and determined that 32 and 1 (rmi-miR-190) miRNA in *I. scapularis *and *R. microplus*, respectively, had counterparts in other species. In addition the expression of three miRNA* sequences were detected (Additional file 3A). As anticipated this approach identified five (rmi-miR-71, rmi-miR-96, rmi-miR-153, rmi-miR-745b and rmi-miR-2001) evolutionary conserved miRNAs in *R. microplus *that were lost in the *D. melanogaster *genome. Overall we identified 51 miRNAs in *R. microplus *that have counterparts in other species. Out of the 51 evolutionary conserved *R. microplus *miRNAs we also detected the expression of 11 miRNA* sequences (Additional file 3A). MiRNA* sequences have been implicated in modifying mature miRNA and 3'UTR evolution in flies [24]. Thus, these species along with the mature tick miRNAs will facilitate the understanding of changes in gene regulatory networks during *R. microplus *life stages and in vital organs.

**Figure 1:**
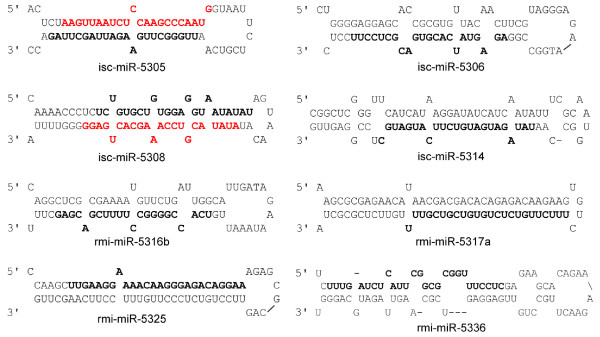
**Precursor miRNA structures for novel tick-specific miRNAs**. Examples of precursor miRNA sequences for novel tick-specific miRNAs are shown. The mature sequences and miRNA* sequences are highlighted in black and red bold fonts, respectively.

In the above analysis, we also identified 36 novel *R. microplus *miRNAs and of these 12 were conserved in the *I. scapularis *genome (Additional file 3B). Examples of pre-miRNAs of novel tick-specific miRNAs showing typical drosha-processed features including localization of mature miRNA within few nucleotides of the loop [25] are shown in Figure 1. Interestingly we detected the expression of miRNA* sequences in four novel tick pre-miRNAs common to both *R. microplus *and *I. scapularis *(Additional file 3B and Figure 1). To determine if the 36 identified novel *R. microplus *miRNAs are restricted to cattle tick, we aligned mature miRNA and pre-miRNAs sequences onto the genomes of *Anopheles gambiae, Apis mellifera, Aedes aegypti, Tribolium castaneum, Nasonia vitripennis, Pediculus humanus, Culex quinquefasciatus *and nine Drosophila genomes (Additional file 4). The mapping coordinates of aligned reads were then used to retrieve genomic segments to evaluate for typical pre-miRNA structures. None of the 36 novel *R. microplus *miRNAs were found conserved in the 16 tested genomes suggesting that these may represent tick-specific miRNAs. The genome size of the cattle tick *R. microplus *is three times larger than that of *I. scapularis*, thus we anticipate that other novel *R. microplus *miRNAs are likely to be identified once the complete genome becomes available.

**Figure 2:**
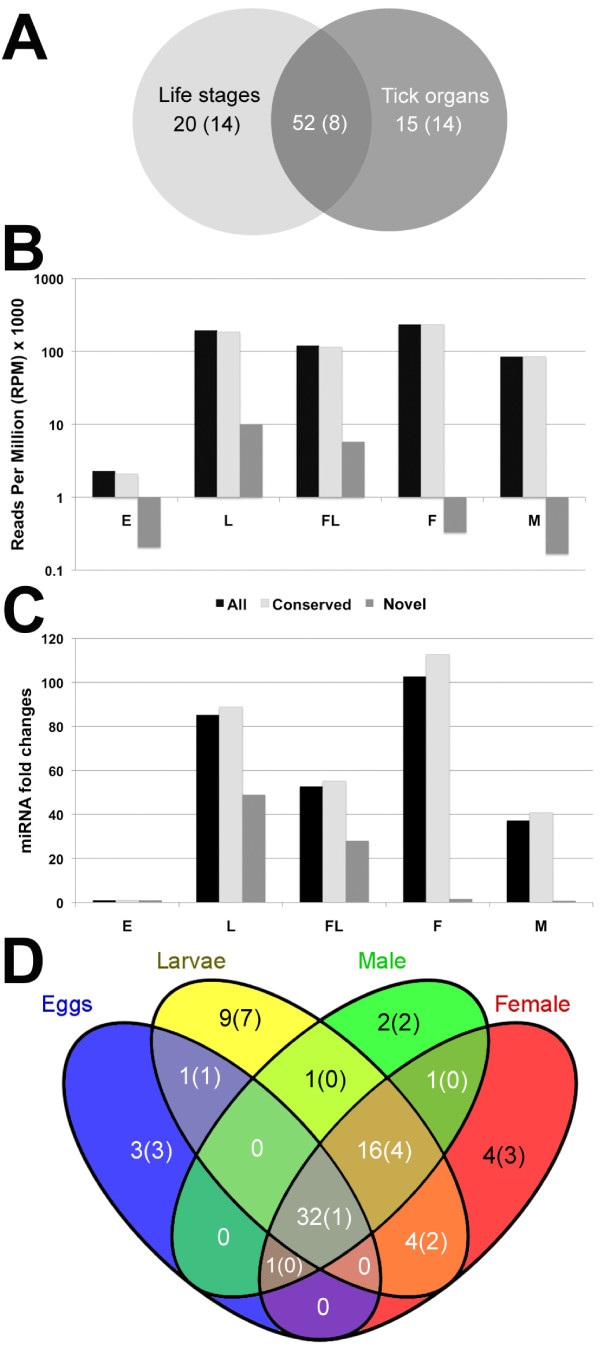
**Global expression changes of evolutionary conserved and novel tick-specific *R. microplus *miRNAs**. A) Overlap of identified *R. microplus *miRNAs found in various life stages and those expressed in adult female tick organs. Values in parentheses correspond to novel tick-specific miRNAs. B) The normalized fraction of small RNA reads (RPM × 1000) overlapping all *R. microplus *miRNAs with known counterparts in other species or all novel tick-specific miRNAs are shown for each life cycle stage. E = eggs, L = larvae, FL = frustrated larvae, F = adult females and M = adult males, respectively. C) Fold-increment in the accumulation of miRNA transcripts in various tick life stages as compared to the egg sample normalized to 1. D) Unique and commonly expressed miRNAs among life stages. Values in parentheses correspond to novel tick-specific miRNAs.

Overall, we identified 87 *R. microplus *miRNAs with 72 of these expressed during various cattle tick life stages and 67 in selected adult female tick organs (Figure 2A and Additional file 3). We found 52 mature miRNAs expressed in both the life stage and tick organ samples with the majority of these (44; 84.6%) corresponding to evolutionary conserved miRNAs (Figure 2A). In contrast, the majority of the 20 life stage-and 15 organ-specific miRNAs corresponded to novel cattle tick-specific miRNAs suggesting that these miRNAs play unique roles at specific life stages and key tick organs. A recent study suggested that miRNA invention is closely related to the evolution of tissue identities in bilaterian species [26]. Further investigation is required to determine if novel tick-specific miRNAs contribute to the implementation of biological features unique to tick species.

Similar to what has been observed in other species we found miRNAs that show expression from both arms of the pre-miRNA. In cattle ticks we observed co-expression of 15 mature and complementary star sequences including rmi-miR-10, rmi-miR-71, rmi-miR-993 and rmi-miR-5308 that are expressed in all cattle tick life stages evaluated in this study (Table 2). Co-expression of rmi-miR-153 and rmi-miR-5314 mature and star sequences was restricted to larval stages while rmi-miR-iab-5p was only detected in adult ticks. These miRNA* sequences were highly conserved between *R. microplus *and *I. scapularis *despite their estimated divergence of 241 ± 28 MYA [1] indicating that these sequences in these species are under selective pressure to avoid nucleotide changes. Interestingly rmi-miR-79*, rmi-miR-281* and rmi-miR-993* are expressed at significantly higher levels than their mature counterparts suggesting that some of the currently annotated mature miRNAs in reference databases may correspond to miRNA* sequences or in some lineages like *R. microplus *miRNA* sequences become the primary transcript from the miRNA/miRNA* duplex.

**Table 2:** *R. microplus *miRNAs expressed from both arms of the precursor

miRNA ID	E miRNA	E miRNA*	L miRNA	L miRNA*	FL miRNA	FL miRNA*	F miRNA	F miRNA*	M miRNA	M miRNA*
rmi-miR-8	8	0	**882**	54	**661**	9	**3,579**	289	**430**	16
rmi-miR-10	11	11	**4,184**	1687	**6,528**	3,140	**2,215**	960	**1,984**	418
rmi-miR-71	**708**	1	0	0	0	0	**6,483**	31	**1,014**	10
rmi-miR-79	29	**61**	191	**1,282**	74	**3,745**	573	**2343**	31	**2031**
rmi-miR-96	0	0	**411**	22	**881**	11	**74**	10	**87**	5
rmi-miR-153	0	0	**42**	7	0	0	0	0	0	0
rmi-miR-307	0	0	0	0	0	0	28	23	0	0
rmi-miR-993	3	**61**	689	**13,722**	213	**9,213**	25	**330**	39	**264**
rmi-miR-2001	**58**	0	**693**	2	**384**	3	**2,953**	7	**271**	3
rmi-miR-iab-4	0	0	24	0	14	0	16	1	9	11
rmi-miR-5305	0	0	**2,245**	35	**1,837**	19	**195**	3	**87**	2
rmi-miR-5308	23	4	**9,115**	261	**9,355**	199	**346**	13	**347**	12
rmi-miR-5313	0	0	3	4	0	0	10	5	0	0
rmi-miR-5314	0	0	66	16	18	4	0	0	0	0

Numbers indicate the total number of reads overlapping mature miRNA and miRNA* sequences. Statistically predominant transcripts (P < 9.62E-06) for each miRNA-miRNA* pairwise comparison in each sample are shown in bold. Abbreviations are as shown in Figure 2B.

### Changes in miRNA expression during cattle tick life cycle stages

To evaluate global changes in miRNA expression during *R. microplus *life stages, short read counts overlapping the 87 miRNAs and 15 miRNA* sequences were normalized as reads per million and compared against the overall expression found in eggs. Our results indicate that in the transition from egg to larval stages there is nearly a 90-fold increase in miRNA transcripts (Figure 2B). Interestingly, larvae exposed to the host for six hours showed a significant reduction in the accumulation of miRNA transcripts as compared to unexposed larvae. The largest accumulation of miRNA transcripts was found in female adults, showing 2.7-fold higher level than males. We also compared changes in expression between evolutionary conserved and tick-specific (unique) miRNAs. Evolutionary conserved *R. microplus *miRNAs are significantly more highly expressed as compared to the identified novel tick-specific miRNAs (Figure 2C), only during larval stages there was an increment in the expression of tick-specific miRNAs. Overall in the life stage samples, we identified 72 *R. microplus *miRNAs, of these 37, 63 and 61 miRNAs were expressed in egg, larval and adult stages, respectively (Figure 2D). Interestingly, 32 (44.5%) of the identified miRNAs were expressed in all cattle tick life stages with 31 of these evolutionary conserved in a range of species and only one rmi-miR-5308 unique to tick species. In contrast most of the stage-specific miRNAs corresponded to novel tick-specific miRNAs. These findings suggest that evolutionary conserved miRNAs play a ubiquitous role through out cattle tick life stages, while most novel tick-unique miRNAs are restricted to specific life stages.

**Figure 3:**
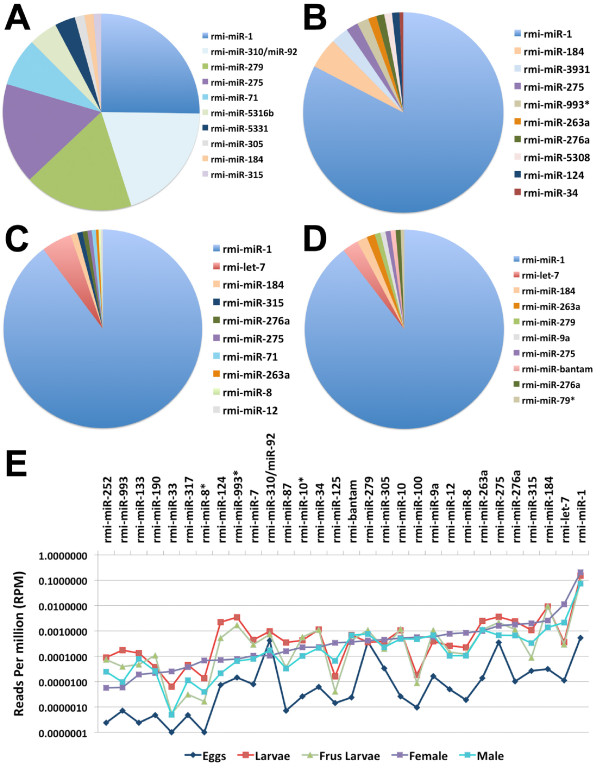
**Top ten most abundant *R. microplus *miRNAs expressed in various cattle tick life stages**. Panels A-D show the percentage expression of the top ten miRNAs for A) eggs, B) larvae, C) adult female, and D) adult male. The number of short reads overlapping a miRNA locus was divided by the total number of reads overlapping all miRNA loci to calculate the percentage of transcripts overlapping each miRNA. Results are show for the top ten most abundant miRNAs in each sample. E) Normalized counts for selected miRNAs expressed in all life stages tested are shown. MiRNA* or miRNA-complementary sequences are denoted with an asterisk.

We next evaluated the percentage fraction of expression of each miRNA in each sample. In eggs there are five abundantly expressed miRNAs including rmi-miR-1, rmi-miR-310/miR-92, rmi-miR-279, rmi-miR-275 and rmi-miR-71 that accounted for 80.6% of the total amount of miRNA transcripts (Figure 3A and Additional file 3), while in all other life stages miR-1 was the most abundantly expressed miRNA (Figure 3). Among the top ten most abundant miRNAs in eggs we found two novel cattle tick-specific miRNAs, rmi-miR-5316b and rmi-miR-5331, that accounted for 4.3% and 3.1% of miRNA transcripts, respectively (Figure 3A). These novel miRNAs were only expressed at statistically significant levels (P < 9.62E-06) during the egg stage (Additional file 5) suggesting they play critical roles during this stage.

Although rmi-miR-1 is the most abundantly expressed miRNA in cattle tick larvae (Figure 3B), we found eight other miRNAs, including rmi-miR-133, rmi-miR-87, rmi-miR-10, and rmi-miR-252, that had a higher fold-change ratio in miRNA transcript levels as compared to that in eggs suggesting that these miRNAs may play a role during larval development (Table 3 and Additional file 6). We also found two novel tick-specific miRNAs, rmi-miR-3931 and rmi-miR-5308, among the top ten expressed larval miRNAs representing 2.7% and 1.2% of the miRNA transcripts (Figure 3B). Interestingly, in Drosophila miR-10 is encoded within the HOX cluster downstream of its target *Sex combs reduced (Scr)*, which is a gene required for proper embryo and adult development in flies [27,28]. It has also been reported that the miR-10 binding site in the *Src *3'UTR is conserved across a large number of arthropod species, some with an estimated divergence time of over hundreds of millions years [28].

**Table 3:** Changes in tick miRNA expression between tick eggs and larvae.

miRNA	Eggs (E)	Larvae (L)	L/E ratio
rmi-miR-133	1	**525**	404.3
rmi-miR-87	3	**1,385**	355.6
rmi-miR-5308	23	**9,115**	305.2
rmi-miR-10	11	**4,184**	292.9
rmi-miR-252	1	**354**	272.6
rmi-miR-124	31	**8,806**	218.8
rmi-miR-184	132	**36,552**	213.3
rmi-miR-bantam	10	**2,746**	211.5
rmi-miR-1	2,242	**602,619**	207.0
rmi-miR-276a	43	**9,443**	169.1
rmi-miR-34	26	**4,494**	133.1
rmi-miR-263	58	**9,836**	130.6

Selected miRNAs showing statistically significant up-regulation are shown in bold (P < 9.61E-06). The L/E expression ratio was calculated using normalized read per million counts for each miRNA. Complete list of miRNAs and statistics are shown in Additional file 6.

Similar to the observation in larval stages, rmi-miR-1 was vastly abundant in adult ticks accounting for 87.4% and 86.2% of miRNA transcripts in females and males, respectively (Figure 3C and 3D). The second most abundant miRNA in adult ticks is rmi-let-7a, a miRNA known to be involved in the transition from late larval to adult stage in worms [29]. It remains to be elucidated if rmi-let-7a is also expressed during tick nymph stage associated with the transition to adult fate. In contrast to egg and larval stages, no novel tick-specific miRNAs were observed among the top ten most abundant miRNAs in adult ticks. These findings suggest that novel tick-specific miRNAs may play key roles during early embryo and larval development stages.

The relative changes in expression of 27 miRNAs and 3 miRNA* expressed in all life stages tested in *R. microplus *are shown in Figure 3E. Interestingly let-7, miR-100 and miR-125 are known to be clustered in the same genomic location in the *D. melanogaster *and *A. gambiae *genomes within 1 kb and 4.5 kb, respectively [30]. In general it is accepted that clustered miRNAs are likely to share highly correlated expression profiles if these are within 50 kb of each other [31]. Our results show that these three miRNAs present similar expression trends (Figure 3E) suggesting that rmi-let-7a, rmi-miR-100 and rmi-miR-125 may also collocate to the same genomic region in the *R. microplus *genome. To validate the observed miRNA expression profiles we conducted real time PCR amplification of five randomly selected miRNAs that showed perfect sequence conservation between ticks and flies. These included rmi-let-7a, rmi-miR-1, rmi-miR-7, rmi-miR-12, and rmi-miR-124. As controls we selected U14 and snoRNA-442 non-coding RNAs, which are reference genes used in Drosophila studies (Ambion, Applied Biosystems), but none of these were either expressed or conserved in cattle ticks. We then normalized the relative expression of all miRNAs in eggs, frustrated larvae and female ticks against the expression of rmi-let-7 in female ticks (Additional file 7). Our results validated the abundant expression of rmi-miR-1 in eggs, frustrated larvae and female samples. We also verified that rmi-let-7 is highly expressed in female ticks as compared to eggs or frustrated larvae samples (Additional file 5). Overall we observed a good agreement between the trends observed in real time PCR quantification and high throughput small RNA sequencing.

### Host-odour recognition triggers changes in tick miRNA expression

*R. microplus *normally parasitizes a single host and it is believed this host specificity depends on the specific detection of bovine phenolic compounds [32]. To evaluate if host odour recognition by ticks may trigger changes in miRNA expression we exposed tick larvae to its host for six hours without allowing the larvae to feed (frustrated larvae), and then we collected small RNA samples for high throughput sequencing. We found 57 miRNA expressed in frustrated larvae, of these 54 were also expressed in unexposed larvae (Additional file 3). There were six miRNAs expressed in unexposed larvae that were not detected in frustrated larvae, particularly rmi-miR-5315 that showed a significant lost in expression (Table 4 and Additional file 8). Only rmi-miR-31a, rmi-miR-285a and rmi-miR-5329 were specifically detected in frustrated larvae, but none of these had significant expression levels (P < 9.62E-05) (Additional file 8). The major difference in miRNA expression found in frustrated larvae, as compared to un-exposed larvae, is the down-and up-regulation of 26 and 23 miRNAs, respectively (P < 9.62E-05) (Additional file 8). Among the most down regulated miRNAs in frustrated larvae are rmi-miR-317, rmi-miR-315, rmi-miR-33, rmi-miR-87 and five tick-specific novel miRNAs including rmi-miR-5306, rmi-miR-5309, rmi-miR-5310, rmi-miR-5312 and rmi-miR-5314 (Table 4 and Additional file 8). Other significantly up-regulated miRNAs in frustrated larvae compared to unexposed larvae are rmi-miR-279, rmi-miR-190, rmi-miR-79*, rmi-miR-96, rmi-miR-5307 and rmi-miR-5308 (P < 9.62E-05) (Table 4 and Additional file 8). The identification of novel tick-specific miRNAs showing up and down-regulation upon exposure to the host make these molecules attractive candidates for further host-recognition functional studies.

**Table 4:** Exposure to host triggers changes in cattle tick miRNA expression.

miRNA	Larvae (L)	Frustrated larvae (FL)	FL/L ratio
rmi-miR-5315	**2,368**	0	0.00
rmi-miR-317	**178**	16	0.13
rmi-miR-315	**4,293**	482	0.16
rmi-miR-87	**1,384**	193	0.19
rmi-miR5306	**566**	93	0.22
rmi-miR-5314	**66**	18	0.38
rmi-miR-993	**690**	214	0.43
rmi-miR-124	**8,807**	2,872	0.45
rmi-miR-5312	**295**	98	0.46
rmi-miR-5309	**231**	99	0.59
rmi-miR-5310	**3,949**	2,643	0.92
rmi-miR-5308	9,115	**9,335**	1.42
rmi-miR-184	36,552	**51,481**	1.94
rmi-miR-10	4,183	**6,527**	2.15
rmi-miR-5307	115	**235**	2.83
rmi-miR-96	412	**882**	2.96
rmi-miR-79*	1,283	**3,746**	4.03
rmi-miR-9a	1,541	**5,835**	5.23
rmi-miR-190	147	**588**	5.49
rmi-miR-279	1,429	**5,919**	5.72

Selected miRNAs showing statistically significant up-regulation are shown in bold (P < 9.62E-05). The FL/L expression ratio was calculated using normalized read per million counts for each miRNA. Complete list of miRNAs and statistics are shown in Additional file 8.

### Changes in miRNA expression between female and male adult ticks

There are significant morphological and behavioural differences between female and male ticks. To evaluate whether there are gender differences in the expression of miRNAs we inspected changes in the miRNA transcriptome between female and male adult ticks. We found 55 miRNAs expressed in adult cattle ticks, of these 46 were expressed in both females and males. We found two miRNAs, rmi-miR-5334 and rmi-miR-5336, specifically expressed in male ticks, while another seven miRNAs were detected in female ticks with four of these also expressed in larval stages (Figure 2D). The three miRNAs uniquely expressed in females were rmi-miR-307, rmi-miR-5317a and rmi-miR-5318, which were expressed at low levels (Additional file 9). Among the miRNAs expressed in both females and males, 22 were up regulated in females (P < 9.62E-05) including the let-7-complex miRNAs rmi-let-7a, rmi-miR-100 and rmi-miR-125 (let-C miRNAs) (Table 5 and Additional file 9). Although similar levels of up regulation of the former two miRNAs in females were observed, rmi-miR-100 showed an expression level similar to that in males. Differential accumulation of these let-7-C miRNAs, which were shown to co-transcribe as a single polycistronic primary transcript [33], may be due to post-transcriptional processing of mature miRNAs from primary transcripts undergoing developmental regulation [34-36]. Interestingly, loss of function of let-7 in Drosophila specifically affects female reproduction, while males retain fertility levels comparable to that in wild type flies [33]. It remains to be elucidated if the observed up regulation of rmi-let-7a in female ticks is required for maintaining both normal fertility and oviposition [33].

**Table 5:** Changes in tick miRNA expression between adult female and male ticks.

miRNA	Female (F)	Male (M)	F/M ratio
rmi-miR-2001	**2,953**	271	10.44
rmi-miR-8	**3,578**	429	7.98
rmi-miR-12	**3,275**	423	7.40
rmi-miR-71	**6,483**	1,014	6.13
rmi-miR-315	**8,471**	1,350	6.01
rmi-let-7	**47,957**	8,650	5.31
rmi-miR-125	**1,436**	267	5.14
rmi-miR-1	**872,047**	298,763	2.80
rmi-miR-276a	**7,574**	2,689	2.70
rmi-miR-275	**6,800**	2,749	2.37
rmi-miR-5305	**195**	87	2.15
rmi-miR-100	**2,403**	1,937	1.19
rmi-miR-310/miR-92	446	**700**	0.61
rmi-miR-279	1,772	**3,103**	0.55
rmi-miR-bantam	1,550	**2,738**	0.54
rmi-miR-133	80	**322**	0.24

Selected miRNAs showing statistically significant up-regulation are shown in bold (P < 9.62E-05). The F/M ratios were calculated using normalized reads per million counts for each miRNA. Complete list of miRNAs and statistics are shown in Additional file 9.

In male ticks we found 9 miRNAs up regulated in comparison to female ticks (P < 9.62E-05) including rmi-miR-133, rmi-miR-bantam, rmi-miR-279 and rmi-miR-310/miR-92 (Table 5 and Additional file 9). It is intriguing to observe the up regulation of two known anti-apoptotic miRNAs, rmi-miR-bantam and rmi-miR-133 in adult males [37,38]. MiR-133 and miR-1 are preferentially expressed in cardiac and skeletal muscles in *Xenopus laevis *[39] both have opposing effects, with miR-1 being rather pro-apoptotic [38]. Interestingly, miR-1 level was shown to significantly increase in response to oxidative stress [38]. In ticks blood digestion has been suggested to be a source of oxidative stress [40,41]. This is particularly relevant for female ticks that ingest large volumes of blood (about 100-fold their own weight) in preparation for the laying of about 2,000 to 3,000 eggs [42], while males rarely feed on blood [43]. Interestingly, apoptosis of salivary glands prior to oviposition in female ticks was reported for *Dermacentor variabilis *[44] and *R. microplus *[45]. Surprisingly *R. microplus *female ticks presented apoptosis just after 24 hours of host detachment [45] contrasting with the 5 days required for *D. variabilis *female ticks [44]. These observations suggest that in *R. microplus *molecular changes are likely to occur prior to female detachment from the host that predispose them for a rapid onset of apoptosis and consequently facilitating egg laying. This notion correlates with the observed significant up regulation of rmi-miR-1 in semi-engorged females as compared to males (Table 5). It remains to be elucidated if blood-mediated oxidative stress in *R. microplus *contributes to the increased expression level of rmi-miR-1 in females and if this miRNA exercise pro-apoptotic activities similar to its *X. laevis *miR-1 counterpart [38] that would ultimately facilitate egg laying.

### microRNA expression in selected tick organs

Primary target organs for cattle tick control are the gut, salivary glands and ovaries of adult female ticks. Nearly 13.3 million short reads were generated for these three organs, of these 790,821 and 5,759 short reads were mapped onto evolutionary conserved and novel tick-specific miRNA loci, respectively (Table 1B). To compare global miRNA expression levels between these samples we normalized all mapped miRNA transcripts in each sample as reads per million. The tick gut sample showed 2-fold and 5-fold higher relative amount of miRNA transcripts as compared to salivary glands and ovaries samples, respectively (Additional file 10A). These differences were due to the larger amount of transcripts mapped onto evolutionary conserved miRNA loci in the gut sample as compared to salivary gland and ovary samples (Additional file 10A). We found 68 miRNAs expressed in all tested tick organs with 50 (6 novel), 50 (9 novel) and 49 (13 novel) expressed in gut, salivary glands and ovaries, respectively (Additional file 10B). A comparison of *R. microplus *miRNAs found in female tick organs with those found in whole semi engorged adult females revealed that 36 miRNAs were commonly expressed in all samples (Additional file 10B). Interestingly the majority of the commonly expressed *R. microplus *miRNAs (94.6%) in tick organs corresponded to known miRNAs in other species, while 18 of the 20 organ-specifically expressed miRNAs found in this study correspond to novel tick-specific miRNAs (Additional file 10C). Most of the novel tick specific miRNAs were expressed at low levels except for rmi-miR-5307 found in all three organs and rmi-miR-3931 present in adult female tick ovaries (Additional file 11).

Similar to what we observed for life stage samples, rmi-miR-1 was the most abundantly expressed miRNA in all sampled organs accounting for ~81-83% of the identified *R. microplus *miRNA transcripts in ovaries and gut, and for 43.1% of the miRNA transcripts in salivary glands (Additional file 11). Other highly expressed miRNAs in salivary glands are rmi-let-7a (15.1%), rmi-miR-275 (10.5%), rmi-miR-263a (5.0%) and rmi-miR-71 (4.2%) (Additional file 11). Recently, two studies have reported the secretion of miRNAs into the saliva [46,47]. Interestingly, among the most abundantly expressed *R. microplus *miRNAs in salivary glands, only let-7 orthologs were found expressed in human salivary glands [46,47]. The expression of let-7 in the salivary glands of ticks and vertebrates suggests a functional role for this miRNA throughout the evolution of this organ.

### Global comparison of miRNA expression in cattle ticks

**Figure 4:**
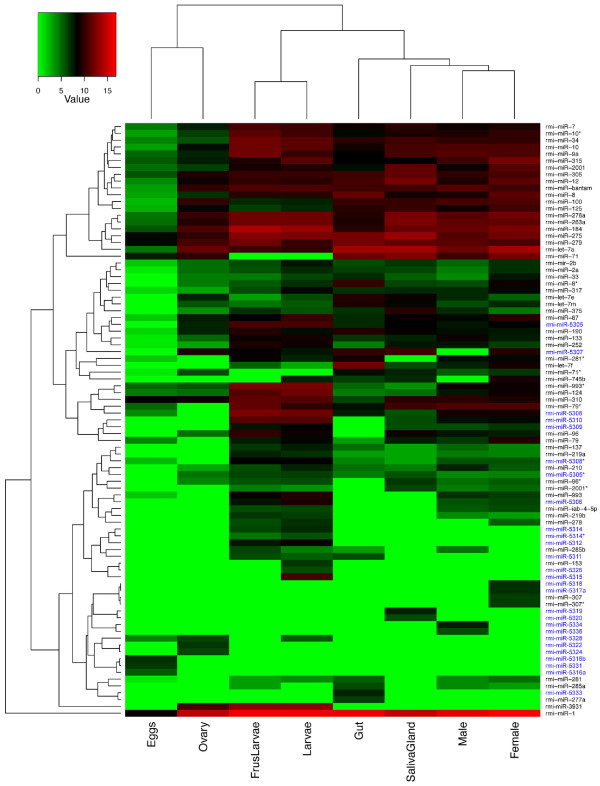
**Clustering of tick samples based on their miRNA expression profiles**. MiRNA counts for each miRNA locus were normalized as reads per million and then log transformed prior to conducting hierarchical clustering as described by Eisen et al. [48]. Novel tick-specific miRNAs are denoted in blue fonts.

To evaluate similarities in the global tick miRNA expression pattern among all samples we conducted a hierarchical clustering as described by Eisen et al. [48] and found that there is low miRNA diversity in eggs, while larvae and female ticks samples showed a large diversity of expressed miRNAs (Figure 4). Adult female tick salivary glands and gut samples clustered together with adult tick samples showing high expression of various miRNAs, but also having unique signatures owing to the presence of tissue-specific miRNAs. Interestingly most of the identified *R. microplus *miRNAs with known counterpart in other species showed high expression levels in most tick life cycle stages and tick organs, while the identified novel tick-specific miRNAs presented more restricted expression patterns accounting for the majority of the tick life stage-specific and tissue-specific *R. microplus *miRNAs.

*D. melanogaster *has 151 annotated miRNAs [15], of these 61 miRNAs are closely located (= < 2 kb) in 17 genomic clusters (Additional file 12). We found tick miRNAs overlapping five of these clusters, including the miR-100:miR-125:let-7, miR-275:miR-305, miR-2a-2:miR-2a-1:miR-2b-2, and miR-310:miR-311:miR-312:miR-313:miR-991:miR-992 clusters (Additional file 12). To infer if these clusters may also be conserved in the cattle tick genome we analysed the co-expression pattern of tick miRNAs across all samples. We observed correlated co-expression profiles of tick miRNAs overlapping two *D. melanogaster *clusters, miR-100:miR-125:let-7 and miR-275:miR-305 indicating that these clusters may also be conserved in the cattle tick genome (Additional file 12).

### Evolutionary conservation of *R. microplus *miRNAs

**Figure 5:**
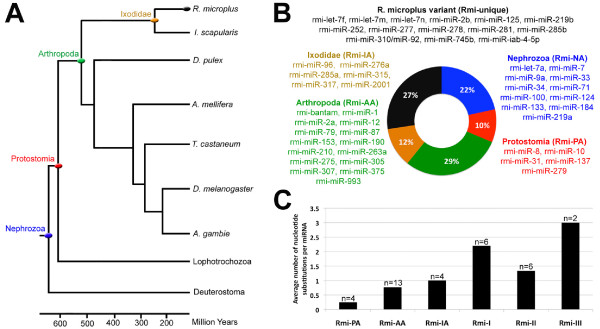
**Evolutionary conservation of *R. microplus *miRNAs**. A) *R. microplus *phylogentic relationships highlighting relevant ancestors are shown. The tree topology was modified from [21]. Diverge times were taken from [16] and [21]; Nephrozoan anecestor = 641-686 MYA; Protostomian ancestor = 618-653 MYA; Arthropoda ancestor = 540MYA. B) Percentage fraction and *R. microplus *miRNAs perfectly conserved since various ancestors are shown as well as those miRNAs representing unique *R. microplus *sequence variants. C) The average number of nucleotide substitutions in miRNAs is shown for the various subsets shown in panel B. MiRNA variants that are unique to *R. microplus *were further divided into three groups as described in the text. Only miRNA for which an ancestral sequence was available were considered, except for Rmi-III where nucleotide changes were compared against known orthologs in insecta species (Additional file 13).

We conducted an evolutionary analysis to determine if miRNAs found in *R. microplus *were perfectly conserved or accumulated nucleotide changes since the last common available ancestor (See Methods). We collected all available miRNAs [15] and for those overlapping the identified *R. microplus *miRNAs we determined the ancestral sequences using a Maximum Likelihood approach [49,50] under the Junkes-Cantor model [51] using a tree topology similar to that shown in Figure 5A (modified from [21]). We found that 73% of the *R. microplus *miRNAs were perfectly conserved since at least 241 MYA with 11, 5, 15 and 6 miRNAs conserved since the Nephrozoan, Protostomian, Arthropoda and Ixodidae ancestor, respectively (Figure 5B). Only 27% of the *R. microplus *miRNAs presented nucleotide substitutions unique to the *R. microplus *lineage. These unique miRNAs can be further divided into three groups: 1) unique miRNA variants in *R. microplus *for which a protostomian ancestral sequence is available (Rmi-I), 2) duplicated copies of *R. microplus *miRNAs (Rmi-II), and 3) unique *R. microplus *miRNAs for which an ancestral sequence is not available and only ortholog sequences in the insecta lineage are known (Additional file 13).

We next examined the number of nucleotide substitutions found in each of the above subsets of *R. microplus *miRNAs by comparing against an ancestral sequence if it can be unambiguously determined (i.e. a *R. microplus *miRNA perfectly conserved since the Arthropoda ancestor was compared against the Protostomian ancestral miRNA sequence or an earlier ancestral sequence if available). In cases where no ancestral sequences are available owing to the gain of that miRNA in a more recent ancestor the number of base substitutions was recorded as zero (i.e. a miRNA that was gained in the Arthropoda ancestor and since then have not accumulated nucleotide substitutions in *R. microplus*). The average number of nucleotide substitutions was then calculated for each subset. We determined 0, 0.25, 0.8 and 1.0 average nucleotide changes for *R. microplus *miRNAs conserved since the Nephrozoan (Rmi-NA), Protostomian (Rmi-PA), Arthropoda (Rmi-AA) and Ixodiade (Rmi-IA) ancestor, respectively (Figure 5C). Furthermore we found 2.2 and 1.3 average nucleotide changes in *R. microplus *miRNA unique variants (Rmi-I) and duplicated miRNAs (Rmi-II) (Figure 5C). Interestingly the average number of nucleotide substitution in miRNAs was directly proportional to the evolutionary time of the ancestor where the oldest conserved miRNAs showed the least amount of nucleotide changes while more recently acquired miRNAs showed slightly increased number of nucleotide changes. Recently, gain of miRNA genes in Metazoan species was suggested to be associated with the increase in morphological complexity [21]. Our findings correlate with the notion that perfectly conserved miRNAs since for example the Nephrozoan ancestor may regulate key basic processes common to a range of animal species and therefore be under stronger selective pressure to remain unchanged throughout the evolution, while those miRNAs more recently acquired or perfectly conserved since a more recent ancestor such the Arthropoda ancestor may play more specific roles for these species.

**Figure 6:**
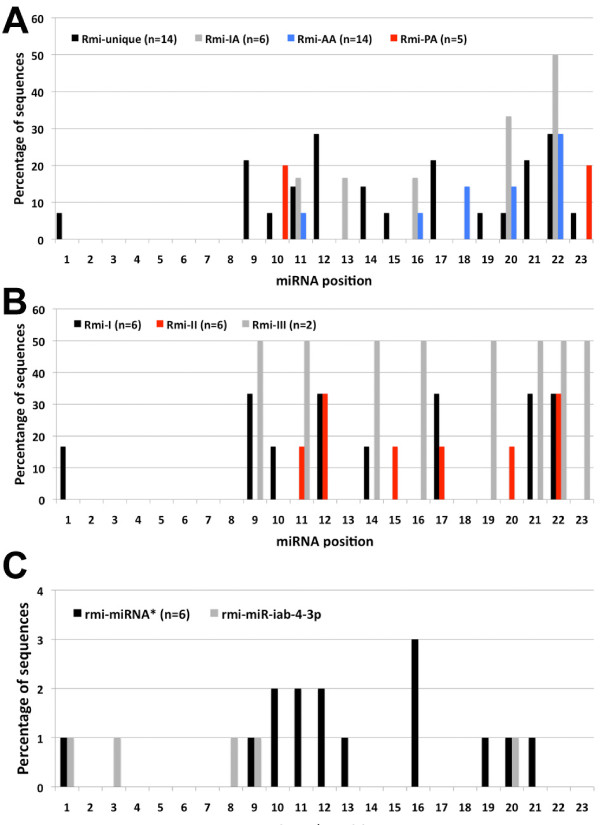
**Mutational profile of *R. microplus *miRNAs as compared to ancestral sequences**. The percentage of nucleotide substitutions from positions 1 to 23 are show for A) subsets of miRNAs defined in Figure 5B; B) unique *R. microplus *miRNA variants subsets as defined in the text; and C) miRNA* sequences showing similar mutational profiles to their mature counterparts with the exception of rmi-miR-iab-4-3p.

We further interrogated the position of the nucleotide substitutions for each of the above subsets as compared to an ancestral sequence and found no base substitutions overlapping the mature miRNA seed (positions 2-8) in all the identified *R. microplus *miRNAs (Figure 6A). *R. microplus *miRNAs conserved since the Protostomian, Arthropoda and Ixodidae ancestor preferentially accumulated nucleotide changes in the 3' end of the mature miRNAs (Figure 6A and Additional file 12). MiRNAs that are unique variants to *R. microplus *particularly those that have a reference Protostomian ancestral sequence or are duplicated miRNAs showed base substitutions in the middle (positions 9 to 12) and 3'end (positions 17 to 23) (Figure 6B). Only unique miRNAs variants without a reference ancestral sequence but with ortholog genes in insecta (Rmi-III) showed an apparent uniform distribution of nucleotide changes from position 9 to 23, but this is likely due to independent accumulated nucleotide substitution in the chelicerata and insecta lineages since the last common Arthropoda ancestor. We also observed that *D. melanogaster *and *A. gambie *tend to have an increased number of base substitutions as compared to *R. microplus *(Additional file 13 and data not shown).

To determine if similar biases in nucleotide substitutions are observed in miRNA* sequences we evaluated nucleotide changes in seven miRNA* (rmi-miR-10*, rmi-miR-281*, rmi-miR-307*, rmi-miR-71*, rmi-miR-8*, rmi-miR-993*, rmi-miR-iab-4-3p) for which ortholog sequences are available among insecta species or in *Lottia gigantea *(Mollusca) for miR-281*. Figure 6C shows that most miRNA* sequences accumulated preferential base substitutions in position 1, in the middle (positions 9 to 12) and the 3'end. Only rmi-miR-iab-4-3p accumulated base substitutions preferentially towards the 5'end, but these nucleotide changes are compensatory mutations to those found in the mature rmi-miR-iab-4-5p sequence (Additional file 13) to maintain a proper pre-miRNA structure (data not shown). Based in our results it is apparent that miRNA* sequences have similar selective pressure to that of mature sequences to avoid base substitutions in the 'miRNA* seed' sequence (positions 2-8) despite this region being complementary to the 3'end portion of the mature miRNA where most base substitutions are accumulated. These findings suggest that *R. microplus *miRNA* sequences may play regulatory roles similar to their mature counterparts and this view is further supported by the significantly higher expression level of rmi-miR-79*, rmi-miR-281* and rmi-miR-993* as compared to their mature miRNA complementary sequences (Additional file 3).

### Seed shifting of *R. microplus *miRNAs

The displacement of the miRNA seed (positions 2-8) towards either the 5' or 3' is referred as 'seed shifting' [21]. As shown in Additional file 13 we found three *R. microplus *miRNAs showing one-nucleotide seed shifting with two of these (rmi-miR-79 and rmi-miR-137) and a third miRNA (rmi-miR-iab-4-5p) displacing the seed towards the 3' and 5', respectively. Interestingly seed shifting in miR-79 was not observed in the sister taxon *I. scapularis *nor the crustacean *Daphnia pulex *with which rmi-miR-79 shares perfect sequence identity. Seed shifting of miR-79 was also found in *Capitella teleta *(Annelida) and insecta species, but not in their sister taxon, namely, Mollusca and Crustacea, respectively. These findings suggest that miR-79 underwent three independent seed shifting events of one-base displacement towards the 3' end in *R. microplus, C. teleta *and insecta ancestor lineages. We also found two independent seed shifting events for miR-137 one in *R. microplus *and another among insecta species (*A. mellifera *and *T. castaneum*) but not in their sister taxon *Daphnia pulex *(Additional file 13). The observed consistency in the seed shifting direction in independent events suggests that this phenomenon may be under selective pressure.

In our data only rmi-miR-iab-4-5p represents an example of seed shifting unique to the *R. microplus *lineage, but this miRNA is an exceptional example as both mature and star sequences have also accumulated a large number of base substitutions (Additional file 13). Despite the observed seed shifting and nucleotide substitutions changes the expression level of the mature rmi-miR-iab-4-5p is 5.2-fold higher than that of rmi-miR-iab-4-3p, which is consistent with the 5.4-fold higher expression level of dme-miR-iab-4-5p as compared to dme-miR-4-3p [15]. These findings suggest that despite the various molecular changes that can take place in a miRNA-miRNA* duplex, the expression ratio at which these molecules are found expressed in distinct taxa remains unchanged reflecting that other mechanisms may regulate the dosage at which each molecule is present to properly control downstream targets.

## Conclusions

This study has identified 87 *R. microplus *miRNAs being 51 of these known in other species including 36 in *I. scapularis*. Overall we found 72 rmi-miRNAs expressed in various cattle tick life stages and 67 rmi-miRNAs expressed in the adult female tick gut, salivary glands and ovaries. Novel tick-specific miRNAs account for the majority of the life-stage and organ-specific expression profiles found in *R. microplus *miRNAs and represent attractive targets for further functional studies. We provide insights into the evolutionary conservation of *R. microplus *miRNAs revealing that the majority of anciently acquired miRNAs remain perfectly conserved, while more recently acquired miRNAs tend to accumulate more nucleotide substitutions in the middle and 3' portion of mature miRNA and miRNA* sequences. Our findings correlates with the notion that long-lived miRNAs are likely to play crucial roles in a wide range of animal species, probably closely related to the acquisition of new organ identities and higher complexity, and therefore be under a stronger selective pressure to remain unchanged as compared to more recently acquired miRNAs.

## Methods

### Tick sampling

The ticks used in this study were obtained from the tick colony (Non-Resistant Field Strain--NRFS) maintained at the Animal Research Institute (Qld Primary Industries & Fisheries), Yeerongpilly, Queensland [52]. Semi-engorged 17-day-old females for the dissections were collected in sterile 5 mL screw top containers (Nalgene, Rochester, NY, U.S.A.) and transferred to the laboratory. Dissections were carried out within the hour after collection. Prior to dissection, the ticks were rinsed with 0.1% DEPC-treated water. Semi-engorged females were fixed on double-sided adhesive tape inside an 8 cm culture dish placed on ice and covered with a few drops of ice-cold sterile PBS. An incision was made with a sterile razor blade just above the right spiracle, starting at the right side of the capitulum and ending the cut at the left side of the capitulum. The dorsal cuticle was then lifted with a pair of dissection tweezers. The salivary gland, midgut and ovary were then removed and homogenized by freezing in liquid nitrogen and ground to powder using sterile RNase ZAP-treated (Applied Biosystems, CA, USA) mortars and pestles. Whole *R. microplus *females (17 days old, n = 10) and males (17 days old, n = 10) collected from the under-side of semi-engorged females were also processed for miRNA extraction. After rinsing in DEPC-treated water, the adult ticks were frozen in liquid nitrogen and subsequently crushed and ground to powder using a sterile mortar and pestle. Approximately 2 g of NRFS strain larvae incubated for 21 days in 1 g batches were obtained for miRNA extractions. One batch of larvae were exposed to the host for 6 hours in a mesh bag attached to the host inside a collar without being able to establish feeding (frustrated larvae). Eggs were also collected into sterile tubes up to 7 days after laying by fully-engorged females. Both larval samples were homogenized by freezing in liquid nitrogen and ground to powder using sterile mortar and pestle. Total RNA and/or enriched small RNA fraction was isolated from the whole adult ticks (male and female 17 days old), larvae, frustrated larvae, eggs and dissected female tick organs using the mirVana microRNA isolation kit as described in the manufacturer's instructions (Ambion, Applied Biosystems).

### RNA isolation

Total RNA or enriched small RNA fractions were prepared from eggs, larvae (unfed and frustrated), adult male and adult female as well as from adult female gut, salivary glands and ovaries. All these samples were ground in liquid nitrogen using a sterile mortar and pestle, and then the RNA was isolated using the mirVana microRNA isolation kit according to manufacture's instructions (Ambion, Applied Biosystems). RNA samples for each condition were collected in triplicate (each as a pool of individuals) and these were kept at -80°C until deep sequencing or real time PCR analysis as described below.

### Small RNA library construction and sequencing

For small RNA library construction and deep sequencing, RNA samples were prepared as follows: for each life cycle stage or tick organ equal quantities (5-7 μg) of enriched small RNA fraction or total RNA isolated from three independent pools as described above were pooled. Approximately 10 μg of enriched small RNA fraction or 20 μg of total RNA representing each life stage or a tick organ were submitted to Illumina/Solexa service provider (GeneWorks, Australia) for sequencing.

In brief, the sequencing was performed as follows: RNA was purified by polyacrylamide gel electrophoresis (PAGE), to enrich for molecules in the range of 18--30 nt, and ligated with proprietary adapters to the 5' and 3'-end termini of the RNA. The samples were used as templates for cDNA synthesis. The cDNA was amplified with 18 PCR cycles to produce sequencing libraries that were subjected to Illumina/Solexa's proprietary sequencing-by-synthesis method.

### Real time PCR amplification

Tick miRNAs identically conserved in *D. melanogaster *including let-7, miR-1, miR-7, miR-12 and miR-124 were selected and TaqMan miRNA assays against these miRNAs were purchased from Applied Biosystems. A total of 10 ng of enriched small RNA fraction for each individual eggs, larvae and female tick samples was used to amplify the miRNAs for up to 50 cycles in a RotoGene 3000 termal cycler.

### Bioinformatics data analysis

Several short read aligners including Maq [53], SOAP [54], RMAP [55], Novoalign http://www.novocraft.com, Bowtie [56] and BWA [57] were selected to evaluate their mapping performance using simulated short reads. 36-bp simulated short reads were generated using MAQ-simulate with mutation rates of 0.1% to up to 16.0%, and introducing read errors using the file simdata/simupar.dat file from the MAQ data installation [53]. Using default parameters of each tool simulated short reads were mapped onto the human and/or the Arabidopsis genomes (Additional file 2). As the originating position of the simulated reads are known the fraction of true positive aligned reads was calculated as [Number of correctly aligned reads/Total number of simulated reads]. Conversely we can also calculate the proportion of false positive aligned reads as [Number of incorrectly aligned reads/Total number of simulated reads]. Based on our results (Additional file 3) we selected Novoalign for aligning our sequenced cattle tick short reads onto the *D. melanogaster *genome using its default parameters http://www.novocraft.com. The *D. melanogaster *(dme_r5.32) genome was downloaded from FlyBase http://flybase.org. We removed short reads mapped with quality alignment scores of zero (Q0), which correspond to reads mapped to two or more locations on the reference genome with identical quality alignment scores. We then conducted a manual inspection of the short reads overlapping known Drosophila miRNA loci to confirm the accuracy and sequence conservation of tick miRNAs aligned onto the Drosophila genome.

To identify novel miRNAs we aligned cattle tick short reads from each sample onto the draft genome of *Ixodes scapularis *(IscaW1.1; 369,459 contigs) and *R. microplus *draft genomic contigs that were recently sequenced and assembled by our group (Bellgard et al. submitted; 175,226 contigs encoding a total of 144,709,321 bp). We then utilized MiRDeep to identify known and novel miRNA candidates as previously described [58]. The identified miRNAs were then compared against 37 known *I. scapularis *miRNAs (miRBase rel. 17.0); matching hits were removed from the novel miRNA candidate dataset. The remaining candidate miRNAs were then subjected to clustering analysis with other known insect miRNAs using MEGA5 [51]. Candidate novel miRNAs with significant similarity to other known miRNAs were removed from the downstream analysis. To assess the conservation of novel miRNAs in other genomes we downloaded from VectorBase http://www.vectorbase.org the genomes of *Anopheles gambiae *(AgamP3), *Aedes aegypti *(AaegL1), *Pediculus humanus *(PhumU1) and *Culex quinquefasciatus *(CpipJ1). The *Nasonia vitripennis *(Nvit_2.0) genome was downloaded from the Nasoria Genome Project http://www.hgsc.bcm.tmc.edu. We downloaded from UCSC Genome Bioinformatics resource http://hgdownload.cse.ucsc.edu the following genomes: *Drosophila pseudoobscura *(dp4), *D. ananassae *(droAna3), *D. erecta *(droEre2), *D. grimshawi *(droGri2), *D. mojavensis *(droMoj3), *D. virilis *(droVir3), *D. willistoni *(droWil1), *D. yakuba *(droYak2), *Tribolium castaneum *(Tcas 2.0) and *Apis mellifera *(Amel_4.0). Novel *R. microplus *miRNA mature and star sequences were then aligned onto all the above genomes using Bowtie [56] and BLAT [59]. Positive hits were further inspected for typical precursor miRNA secondary structure using RNAfold [60].

### Statistical analysis

Statistically significant changes in miRNA expression between samples was calculated using four statistical tests including Pairwise Audic & Claverie test (AC), Pairwise Chi sq. test (Chi2 × 2) and Multiple Chi sq. test (Chi) as previously described by Romualdi et al. [61]. Bonferroni correction was applied to the data and significant thresholds of 9.62E-05 or 9.62E-06 were defined depending on the comparisons.

### Evolutionary analysis

We conducted an evolutionary analysis to determine if miRNAs found in *R. microplus *were perfectly conserved or accumulated nucleotide changes since the last common ancestor based on available sequences for each miRNA (miRBase release 17). Multiple sequence alignments for each miRNA were generated using ClustalW [62] within the MEGA5 package [51]. Ancestral sequences were determined using a Maximum Likelihood method [49,50] under the Junkes-Cantor model [63] using a tree topology similar to that shown in Figure 5A (modified from [21]). We then compared each *R. microplus *miRNA sequence against the respective inferred ancestral sequence (Additional file 13) and determined if each *R. microplus *miRNA was perfectly conserved since the Nephrozoan, Protostomian, Arthropoda or Ixodidae ancestor or if it represented a unique nucleotide variant in the *R. microplus *lineage (Figure 5A and 5B). This resulted in a classification of each miRNA into one of the following categories: 1) conserved since Nephrozoan ancestor (Rmi-NA), 2) conserved since Protostomian ancestor (Rmi-PA), 3) conserved since Arthropoda ancestor (Rmi-AA), 4) conserved since Ixodidae ancestor (Rmi-IA), or 5) unique variant to *R. microplus *(Rmi-unique).

## Authors' contributions

RB contributed with the conception, bioinformatics, experimental validations, evolutionary analysis, data analysis and interpretation, and writing of the manuscript. GKG and PM contributed with the bioinformatics data processing. BZ contributed with the collection of samples for deep sequencing. KI, YT and TG provided critical feedback on the evolutionary analysis. ALT, FDG and MB provided critical review of the manuscript and interpretation of data. All authors read and approved the final manuscript.

## Supplementary Material

Additional file 1 **Supplemental Methods**. Click here for file

Additional file 2 **Single-end mapping performance of short read aligners**. A) The percentages of correctly mapped reads at the indicated mutation rates are shown for each tool. For each point 70,000 short reads in triplicate were mapped. B) The percentages of incorrectly aligned reads at the indicated mutation rates are shown for each tool. For each point 70,000 short reads were mapped in triplicate. Click here for file

Additional file 3 **Evolutionary conserved and novel tick-specific *R. microplus *miRNAs expressed during various life stages and selected organs**. *Rhipicephalus microplus *miRNAs with known counterpart in *D. melanogaster*, *I. scapularis *or other relevant species are shown with reference miRBase IDs. The number of short reads overlapping each miRNA locus, the corresponding percentage fraction of the total number of reads overlapping all miRNA loci for each sample and the normalized reads per million (RPM) counts for each miRNA in each sample are shown. N.A. = Not available in miRBase and/or not cloned previously. Click here for file

Additional file 4 **Mapping of novel *R. microplus *miRNAs on various genomes**. Novel *R. microplus *miRNAs were aligned onto various genomes using Bowtie allowing up to two mismatches. Regions where miRNAs aligned were extracted the typical pre-miRNA hairpin structure evaluated using RNAfold [60]. Additionally, identified pre-miRNAs in *R. microplus *and *I. scapularis *genomic contigs were aligned on the selected genomes using BLAT. No miRNA counterparts for the novel *R. microplus *miRNAs were identified in the current available genome assemblies for the 16 tested species. Genome assemblies for each species are indicated in parentheses. N.F. = Not found. Click here for file

Additional file 5 **Statistically significant differences in miRNA expression among *R. microplus *life stage samples**. Statistically significant changes in miRNA expression between libraries was calculated using Pairwise Audic & Claverie (AC), Pairwise Chi sq. (Chi2 × 2) and Multiple Chi sq. test (Chi) tests as previously described by Romualdi et al. (2003). Bonferroni correction was applied and significant thresholds of 9.62E-06 for AC and Chi2 × 2, and 9.62E-05 for Chi were defined. Bold fonts denote values that are statistically significant. Click here for file

Additional file 6 **Comparison of changes in tick miRNA expression between egg and larval stages**. Statistically significant changes in miRNA expression between libraries was calculated using six statistical tests including Pairwise Audic & Claverie test (AC), Pairwise Chi sq. test (Chi2 × 2) and Multiple Chi sq. test (Chi) as previously described by Romualdi et al. (2003). Bonferroni correction was applied and significant thresholds of 9.61E-06 for AC, Chi2 × 2 and Chi were defined. Bold fonts denote values that are statistically significant. Click here for file

Additional file 7 **Real Time PCR quantification of selected *R. microplus *miRNAs**. Cattle tick miRNAs identically conserved in *D. melanogaster *were selected and specific Drosophila miRNA TaqMan probes (Applied Biosystems) were used to amplify these in eggs, larvae and female tick samples. Click here for file

Additional file 8 **Comparison of changes in tick miRNA expression between larvae and frustrated larvae samples**. Statistically significant changes in *R. microplus *miRNA expression between libraries was calculated using three statistical tests including Pairwise Audic & Claverie test (AC), Pairwise Chi sq. test (Chi2 × 2) and Multiple Chi sq. test (Chi) as previously described by Romualdi et al. (2003). Bonferroni correction was applied and significant thresholds of 9.62E-05 for AC, Chi2 × 2 and Chi was defined. Bold fonts denote values that are statistically significant. RPM = Reads per million; N.A. = Not applicable. Click here for file

Additional file 9 **Comparison of changes in tick miRNA expression between female and male tick samples**. Statistically significant changes in miRNA expression between libraries was calculated using four statistical tests including Pairwise Audic & Claverie test (AC), Pairwise Chi sq. test (Chi2 × 2) and Multiple Chi sq. test (Chi) as previously described by Romualdi et al. (2003). Bonferroni correction was applied and significant thresholds of 9.62E-05 for AC, Chi2 × 2 and multiple Chi was defined. Bold fonts denote values that are statistically significant. Click here for file

Additional file 10 **Global comparison *R. microplus *miRNAs expressed in selected adult female tick organs**. A) The normalized fraction of small RNA reads (RPM × 1000) overlapping all *R. microplus*miRNAs with known counterparts in other species or all novel tick-specific miRNAs are shown for each life stage. F = adult female ticks, Gu = Gut, SG = Salivary glands and Ov = Ovaries. B) Unique and commonly expressed miRNAs among adult female tick samples. Values in parenthesis correspond to novel tick-specific miRNAs. Click here for file

Additional file 11 **Statistically significant differences in miRNA expression among tick adult samples**. Whole individuals and selected organs derived from adult female ticks are compared. Statistically significant changes in miRNA expression between libraries was calculated using Pairwise Audic & Claverie (AC), Pairwise Chi sq. (Chi2 × 2) and Multiple Chi sq. test (Chi) tests as previously described by Romualdi et al. (2003). Bonferroni correction was applied and significant thresholds of 1.60E-05 for AC and Chi2 × 2, and 1.60E-05 for Chi were defined. Bold fonts denote values that are statistically significant. MiRNAs with at least one pairwise comparison with a statistically significant difference are shown. N.A. = Not applicable, no statistical significant differences. Click here for file

Additional file 12 **Expression of tick miRNAs overlapping Drosophila miRNAs organized as clusters**. Drosophila miRNAs located within 2 kbp are grouped within the same cluster. Red font = Tick miRNAs that show correlated co-expression patterns across all tick samples. Black font = Expressed tick miRNAs that do not show a correlated co-expression pattern. Grey font = denote miRNAs not expressed in the sequenced tick samples in this study. Click here for file

Additional file 13 **Evolutionary analysis of *R. microplus *miRNAs**. Evidence supporting *R. microplus *miRNAs conservation since the A) Nephrozoan, B) Protostomian, C) Arthropoda and D) Ixodidae ancestors are presented as well as E) miRNA nucleotide variants unique to *R. microplus *based on currently available datasets (miRBase release 17). Ancestral sequences were determined using a Maximum Likelihood method [49,50] under the Junkes-Cantor model [63] using a tree topology similar to that shown in Figure 5A (modified from [21]). Evolutionary analyses were conducted in MEGA5 package [51]. Key miRNAs supporting each ancestral state were selected. Base-substitutions compared to predict ancestral sequences are highlighted in red fonts. Green fonts denote bases for which an ancestral state could not be determined. *R. microplus *miRNAs are denoted by bold fonts. Click here for file
