# Supplementary material for: Evolutionary conserved microRNAs are ubiquitously expressed compared to tick-specific miRNAs in the cattle tick Rhipicephalus (Boophilus) microplus
Source: BMC Genomics. 2011 Jun 24;12:328. doi: 10.1186/1471-2164-12-328 (PMC3141673; doi:10.1186/1471-2164-12-328)
Supplement: Additional file 1 — Supplemental Methods. [file 1471-2164-12-328-S1.DOC]

# Additional file 1 - Supplementary Methods

## Simulated Data

The MAQ SIMULATE command was used as the main program for generating simulated Illumina reads[1]. MAQ SIMULATE by default produces Sanger FASTQ format for all simulated short reads. A sequence length of 36 bp was used for all benchmarking simulations. The unmasked human chromosome 22 (NCBI36 version) was used as the base reference sequence for all mutation/insertion/deletion simulations.

## Single-End

In the single end (SE) evaluation paired-end reads were simulated and the two files were concatenated to produce a file of single end reads. As the human reference genome contains some unfinished regions that are coded as blocks of N's we removed simulated paired reads if either of end contained two or more “N” IUB codes. The raw-36-simupar.dat file (available in the MAQ-data package [http://maq.sourceforge.net](http://maq.sourceforge.net/)) was used to train quality values for all simulated reads.

100,000 Read pairs were simulated on human chromosome 22 at incremental mutation rates that covered a gradient of 0.1%-16% randomly introduced mutations rates, allowing 30% of mutations to be insertions/deletions, with a 0.3 probability of further extending an insertion/deletion to greater than 1 base pair. The indel extension probability feature was a modification to the existing “MAQ SIMULATE” command. All programs evaluated were subsequently run on these datasets using the original unmasked human chromosome 22 sequence as the target database.

Output of all programs tested were converted to pairwise alignment PAF format and evaluated using the “paf_utils.pl pafeval” script in the MAQ package[1].

Parameter options used for each tool are show in the following Table:

| **Tool [Reference]** | **Version** | **Parameters** | **URL** |
| --- | --- | --- | --- |
| SHRiMP[2] | 1.05 | default; probcalc P=0.5 | <http://compbio.cs.toronto.edu/shrimp/> |
| MAQ[3] | 0.6.8 | default | [http://maq.sourceforge.net](http://maq.sourceforge.net/) |
| Novoalign/  Novopaired [4] | 1.04 | default | [www.novocraft.com](http://www.novocraft.com/) |
| RMAP[5] | 0.41 | -m 4 -w 36 -v | <http://rulai.cshl.edu/rmap> |
| ELAND[6] | GA Pipeline-0.3.0b3 | default | NA |
| SOAP[7] | 1.03 | -c 42 -r 0 -g 3 | [http://soap.genomics.cn](http://soap.genomics.cn/) |

## Paired-End

MAQ and Novoalign-PE were used for the paired-end evaluations because these tools were scalable to searching the human genome with paired-end sequences using a single CPU. 2,000,000 Reads (1000,000 pairs) were simulated from human chromosome 22 in the same manner as for single-end runs with the major difference being that paired-end files were not concatenated together. “N” characters were filtered as with the single-end simulations. A mutation gradient of 0-1% was used at increments of 0.2% with a 1% indels and the probability of extending an indel set to 0.3.

## Paired-End Indel Dataset

In order to test the efficiency of indel detection by short read aligners using paired-end sequences, simulated reads were generated in the same manner as for the single-end method. A major difference was that the MAQ SIMULATE command was used without extension of any insertions/deletions. 3,000,000 (1.5M pairs) reads were simulated as before on chromosome 22 at mutation rates of 0.1%, 0.5%, 1% 2%, 6%, 10% and 12% while the probability of an insertion or deletion was increased previously from 0.3 to 0.5. The target database consisted of human chromosomes 21 and 22 [3].

MAQ and Novoalign-PE were initially run on all datasets and converted to the binary .map format used by the MAQ package[1]. The MAQ INDELPE command was subsequently used to call insertions/deletions from these results using the information of known SNP locations produced by the MAQ SIMULATE command.

## Real Data

The performance of alignment tools was assessed using data available in the NCBI trace archive. We selected a set of Illumina Genome Analyzer paired-end reads in from the Human genome sequencing of an African male individual (HapMap: NA18507) [refs <http://www.hapmap.org/citinghapmap.html.yo>]. The 200x36x37-071207_EAS51_0064-s_2_1.fastq and 200x36x37-071207_EAS51_0064-s_2_2.fastq FASTQ-formatted files were downloaded from the <ftp://ftp.ncbi.nih.gov/pub/TraceDB/ShortRead/SRA000271/fastq/> FTP site.

The first 500,000 short reads from 200x36x37-071207_EAS51_0064-s_2_1.fastq were chosen for the single-end evaluation while the whole set inclusive of the two files were used in the paired-end runs.

**References**
