# Supplementary figures and images for: Evolutionary conserved microRNAs are ubiquitously expressed compared to tick-specific miRNAs in the cattle tick Rhipicephalus (Boophilus) microplus
Source: BMC Genomics. 2011 Jun 24;12:328. doi: 10.1186/1471-2164-12-328 (PMC3141673; doi:10.1186/1471-2164-12-328)

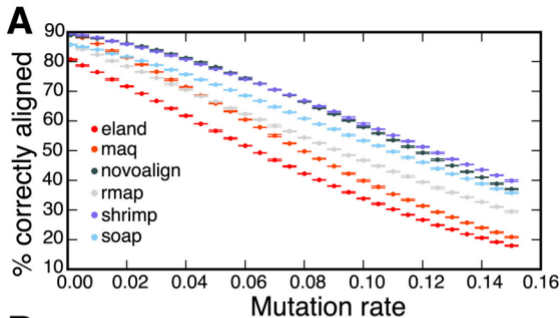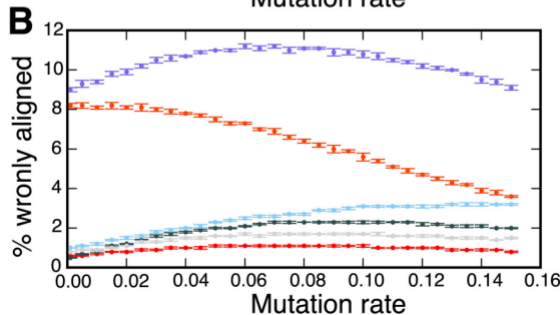

Supplement: Additional file 2 — Single-end mapping performance of short read aligners. A) The percentages of correctly mapped reads at the indicated mutation rates are shown for each tool. For each point 70,000 short reads in triplicate were mapped. B) The percentages of incorrectly aligned reads at the indicated mutation rates are shown for each tool. For each point 70,000 short reads were mapped in triplicate. [file 1471-2164-12-328-S2.PDF]
